# Macromolecular Drug Carriers for Targeted Glioblastoma Therapy: Preclinical Studies, Challenges, and Future Perspectives

**DOI:** 10.3389/fonc.2018.00624

**Published:** 2018-12-17

**Authors:** Drazen Raucher, Sonja Dragojevic, Jungsu Ryu

**Affiliations:** Department of Cell and Molecular Biology, University of Mississippi Medical Center Jackson, MS, United States

**Keywords:** macromolecular, glioblasoma, preclinic study, drug carriers, targeted therapies

## Abstract

Glioblastoma, the most common, aggressive brain tumor, ranks among the least curable cancers—owing to its strong tendency for intracranial dissemination, high proliferation potential, and inherent tumor resistance to radiation and chemotherapy. Current glioblastoma treatment strategies are further hampered by a critical challenge: adverse, non-specific treatment effects in normal tissue combined with the inability of drugs to penetrate the blood brain barrier and reach the tumor microenvironment. Thus, the creation of effective therapies for glioblastoma requires development of targeted drug-delivery systems that increase accumulation of the drug in the tumor tissue while minimizing systemic toxicity in healthy tissues. As demonstrated in various preclinical glioblastoma models, macromolecular drug carriers have the potential to improve delivery of small molecule drugs, therapeutic peptides, proteins, and genes to brain tumors. Currently used macromolecular drug delivery systems, such as liposomes and polymers, passively target solid tumors, including glioblastoma, by capitalizing on abnormalities of the tumor vasculature, its lack of lymphatic drainage, and the enhanced permeation and retention (EPR) effect. In addition to passive targeting, active targeting approaches include the incorporation of various ligands on the surface of macromolecules that bind to cell surface receptors expressed on specific cancer cells. Active targeting approaches also utilize stimulus responsive macromolecules which further improve tumor accumulation by triggering changes in the physical properties of the macromolecular carrier. The stimulus can be an intrinsic property of the tumor tissue, such as low pH, or extrinsic, such as local application of ultrasound or heat. This review article explores current preclinical studies and future perspectives of targeted drug delivery to glioblastoma by macromolecular carrier systems, including polymeric micelles, nanoparticles, and biopolymers. We highlight key aspects of the design of diverse macromolecular drug delivery systems through a review of their preclinical applications in various glioblastoma animal models. We also review the principles and advantages of passive and active targeting based on various macromolecular carriers. Additionally, we discuss the potential disadvantages that may prevent clinical application of these carriers in targeting glioblastoma, as well as approaches to overcoming these obstacles.

## Introduction

**Figure 1:**
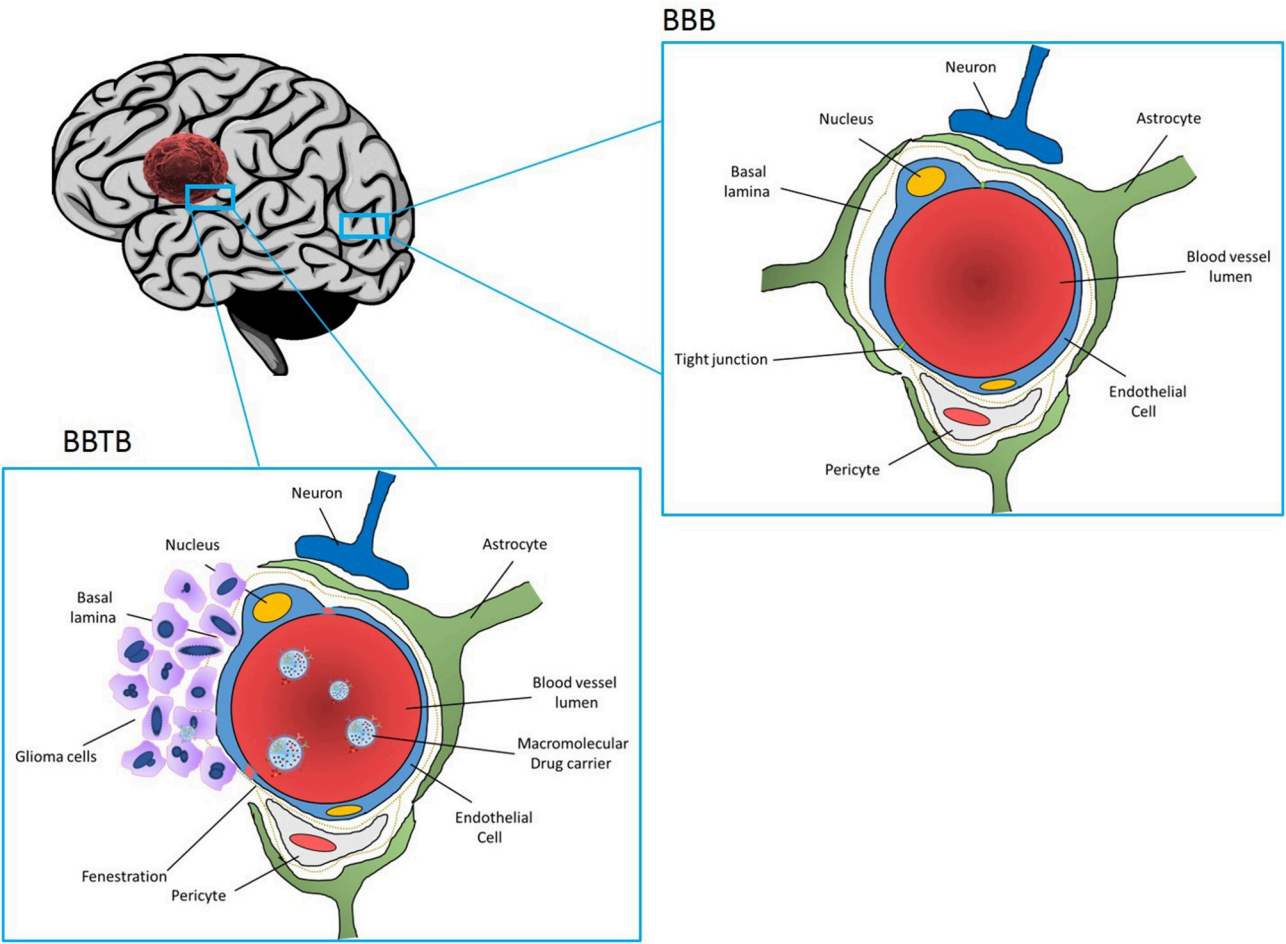
Section of the brain showing normal part of the brain and blood brain barrier (BBB) (upper figure), and part of the brain infiltrated with GBM Blood vessels are enclosed by endothelial cells connected with tight junctions which seal the intercellular space and prevent entrance of drugs from the blood stream to the brain. BBB is further reinforced with a basal lamina. While basal lamina is disrupted by GBM tumor cells (figure the left), endothelial cells are still present forming blood brain tumor barrier (BBTB). Nanoparticles loaded with drugs and coated with receptors specific to the tumor vasculature and glioma cells can interact with the BBTB allowing for transcytosis and delivery of the drugs to GBM tumor cells.

Glioblastoma (GBM) is the most common and the most aggressive primary malignant tumor of the central nervous system. Current therapy regimens are initial surgical resection which is followed by radiation and chemotherapy using the DNA alkylating agent Temozolomide. However, glioblastoma tumors are very aggressive and resistant to multimodal therapies, and the average life expectancy and overall survival is < 18 months. Therefore, current clinical therapies are ineffective as they are more palliative in nature than curative. Treatment options are limited since complete surgical resection is impossible and since tumor tissue is heterogeneous and penetrates surrounding healthy brain tissue. As a result, almost all the patients develop recurrent tumors, which are more aggressive and often resistant to anticancer drugs. Furthermore, drug delivery to the brain is hampered by the presence of blood brain barrier (BBB) (Figure 1), resulting in poor delivery of drugs to the tumor tissue and dose related systemic toxicity in healthy tissues. Considering limitations and overall ineffectiveness of the current approaches in the treatment of glioblastoma, there is an urgent need for more efficient treatments to achieve improved outcome and increase overall survival in glioblastoma patients.

One of the approaches of tumor specific drug delivery is based on macromolecular drug carriers. Advantages of macromolecular carriers over small molecule drugs include protection of the drugs from degradation, improvement of drug solubility, and blood plasma half-life-time, release of the drugs in the optimal dosage range, and delivery of the anticancer agents specifically to the tumor. Currently used macromolecular drug delivery systems, such as liposomes and polymers, passively target solid tumors by capitalizing on abnormalities of the tumor vasculature, its lack of lymphatic drainage, and the enhanced permeation and retention (EPR) effect. However, to achieve therapeutic efficacy in treating GBM, polymeric carriers must successfully overcome several transport barriers (including BBB), extravasate tumor micro vessel walls, and penetrate the plasma membrane of the tumor cells. In addition to passive targeting, further selectivity of macromolecules can be achieved by active targeting. Active targeting approaches include the application of cancer biomarker proteins that bind to overexpressed cell surface proteins in specific cancer cells. It also includes stimuli-responsive macromolecular carriers which can release anticancer drugs specifically in the tumor tissue or tumor cells in response to internal or external stimuli. Internal stimuli drug release is based on the fact that tumor tissue has a different environment compared to normal tissue; more acidic pH, higher redox potential, and/or overexpressed proteins, and enzymes. In addition, stimuli such as light, ultrasound, a magnetic field, and temperature, can be also applied to the tumor site externally to allow drug to be released and their molecular target in the cancer cells reached.

In the present review, we report the use of macromolecular carriers with different composition, including lipids, proteins, and synthetic nanoparticles and we consider their targeting aspects. We also review selected preclinical brain drug delivery macromolecular carriers and highlight their potential in the clinical treatment of glioblastoma.

## Active Targeting

Active targeting to tumor sites generally exploits an intrinsic cell characteristic to obtain drug delivery. Utilizing a homing device such as an antibody or ligand, a drug can bind tumor cells through antigens or receptors without affecting any other normal tissues (1).

This classic concept of active targeting has been successful due to its high selectivity and binding affinity to produce a series of antibody-drug conjugates or ADC. Currently, there are several marketed ADC including Brentuximab and Trastuzumab; however, much more promising ADC are under investigation in clinical trials (2). Despite all of the enthusiasm toward this type of approach, ADC strategy is also facing a few problems that must be resolved in order to take a greater step forward. These problems include low efficiency in cellular uptake or in endosomal escape, heterogeneity of tumor cells in the expression of specific receptors, and challenges in manufacturing (3).

Another example of active targeting is stimuli-responsive targeting. As knowledge of tumor biology and technologies advances, a variety of novel, and smart devices have been introduced showing unprecedented efficiency of drug delivery. Environmentally responsive macromolecular drug carriers can release cargo drugs in the targeted tumor tissues as a response of external stimuli such as heat, light, ultrasound, and a magnetic field. This triggered drug release provides advantages over other types of active targeting technologies in that it allows exquisite control over time and location of drug release (4).

### Receptor-Mediated Endocytosis

Clathrin-dependent endocytosis is known for a predominant mechanism for the internalization of ADC even though the other pathways including caveolin-mediated, clathrin–caveolin-independent, and cholesterol/macropinocytosis-mediated are also reported in literature (5). To briefly describe this process, once binding to specific receptor, ADC-receptors are invaginated by cells through the formation of clathrin-coated vesicles. With dynamin GTPase, the vesicles are then released from the membrane with some of the mature vesicles fusing with lysosomes to form lysosome-late-endosome hybrids through Ras-related protein 7 (Rab7). While cells perform this whole procedure for acceptance of the ADC-receptors, ADC have a couple of opportunities to release drugs from the antibody. First, ADC can release drugs in the endosomal phase. Acid-labile linkers such as (6-maleimidocaproyl) hydrazine (EMCH) allow ADC to unload drugs in the endosome because of the acidic environment of endosomes. Second, ADC can release drug in the lysosome. The high content of enzymes such as cathepsins and collagenases in lysosomes can digest some dipeptide linkers such as valine-citrulline (vc) or phenylalanine-lysine linkers which are specific for cathepsin B. Third, even without a cleavable linker between antibody and drug, drugs can be released by proteolytic digestion in the lysosome. Some products from the digestion of metabolites still retain the original activities of the drug and express their activities in the cells or neighboring cells; thebystander effect (6).

Another mechanism involved in internalization of ADC-receptor is autophagy. As a part of the autophagy process, ADC-receptor can be taken up by autophagosomes and digested in autolysosomes releasing drugs afterward (7).

### Antibodies

#### Epidermal Growth Factor Receptor EGFR

The most common genetic aberration associated with malignant glioma is amplification of the epidermal growth factor receptor, with a frequency of about 50% (8).

Targeting the receptor for epidermal growth factor receptor (EGFR) has been rewarding in cancer and many pharmaceuticals are approved alone or in combination with chemotherapy for colorectal cancer, non-small-cell lung cancer, and pancreatic cancer, among others, but not for gliomas (9). It remains unresolved why EGFR targeting has not been successful for glioma as it should be ideally suitable in the context of this disease (9).

Jamali et al. delivered curcumin using Poly (D, L-lactic-co-glycolic acid) nanoparticles (PLGA NPs). Monoclonal antibody targeting epidermal growth factor receptor variant III (EGFRvIII) was incorporated into PLGA NPs showing selective internalization of the NPs by an EGFRvIII overexpressed human glioblastoma cells and increased photodynamic toxicity of curcumin (10).

In another study, etoposide (ETP) was loaded in solid lipid nanoparticles (SLNs) containing a monoclonal antibody for insulin receptors and another monoclonal antibody against EGFR (11). Since insulin receptors are found on human brain microvascular endothelial cells (HBMEC), these dual targeting nanoparticles passed across HBMEC/HA (human astrocytes), an *in vitro* model for blood-brain barrier, and increased cytotoxicity in the treatment of U87MG cells (Table 1).

**Table 1:** Active targeting with antibodies (or ligands) for GBM treatment.

**Group**	**Carrier/Technology**	**Targeting method**	**Cargos**	**References**
Nanoparticle	Poly (D, L-lactic-co-glycolic acid), PLGA	Anti-EGFRvIII (A-EGFRvIII-f)	Curcumin	(11)
	PLGA	OX26 type monoclonal antibody for transferrin receptor	Temozolomide	(12)
	Bovine serum albumin-polycarprolactone (BSA-PCL)	Anti-EGFR	Radioiodine	(13)
	(PLGA) and PLGA-polyethylene glycol (PLGA-PEG) polymers	Anti-Fn14 receptor		(14)
	PEGylated-hydrophilic carbon clusters	Epidermal growth factor receptor (EGFR) binding peptide		(15)
	Superparamagnetic iron oxide nanoparticle (SPION) based polymeric nanocomposites	Antibody against nestin, a stem cell marker, and transferrin	Temozolomide	(16)
	PLGA	Human/mouse chimeric anti-GD2 antibody ch14.18/CHO, enabling specific targeting of GD2-positive GBM cells	Letrozole,	(17)
	PEG-PE-based polymeric micelles	The micellar system was decorated with GLUT1 antibody single chain fragment variable (scFv)	Doxorubicin, Durcumin	(18)
	Magnetite particles + PEG	Plant lectin viscumin		(19)
	non-living bacterially-derived minicells	Epidermal growth factor receptor (EGFR) targeting	Doxorubicin	(20)
	Nanorings made of dihydrofolate reductase (DHFR) fusion proteins	A PEGylated EGFR targeting peptide (LARLLT)	Methotrexate	(21)
	Graphene oxide (NGO)	Integrin avß3 monoclonal antibody (mAb)	Pyropheophorbide-a	(22)
	Solid lipid nanoparticles (SLNs)	83–14 Monoclonal antibody and anti-epithelial growth factor receptor	Etoposide (ETP)	(11)
	SLN	Melanotransferrin antibody and tamoxifen	ETP	(23)
	SLN	melanotransferrin antibody	ETP	(24)
	superparamagnetic iron nanoparticles (SPION)	Hsp70-specific antibody (cmHsp70.1)		(25)
	polylysine-DTPA (Diethylenetriamine pentaacetate)	Monoclonal antibody to Connexin 43	Gd(III)	(26)
	iron oxide nanoparticles (IONP)	Anti-EGFRvIII-cetuximab (an EGFR- and EGFRvIII-specific antibody		(27, 28)
	Bovine serum albumin	Monoclonal antibodies against vascular endothelial growth factor (VEGF)	Ferric oxide (Fe_3_O_4_) as a MRI contrast agent	(29)
	nanoparticles	Fn14 monoclonal antibody		(30)
	Nanogels based on PEG and polymethacrylic Acid block copolymer (PEG-b-PMAA)	Monoclonal antibodies to connexin 43 (Cx43)	Cisplatin	(31)
	Activatable cell-penetrating peptides (ACPP) : cyclic-RGD PLGC(Me)AG-MMAE-ACP	Integrin a(v)ß(3)-binding domain, cyclic-RGD, was covalently linked to the ACPP	Monomethyl-lauristatin E (MMAE)	(32)
	Nanogels (PEG-b-PMAA) diblock copolymer base)	Monoclonal antibodies to connexin 43 and brain-specific anion transporter (BSAT1)	Cisplatin	(33)
Liposome	liposomes	A chlorotoxin peptide fused to human IgG Fc Region without hinge sequence (M-CTX-Fc) targeting CD44	Doxorubicin	(34)
	immunoliposome	Angiopep-2 (An2) and anti-CD133 monoclonal antibody (CD133 mAb)	Temozolomide	(35)
	Lipid nanocapsule	Antibody for CXCR4	Rhenium-188	(36)
	nanometric liposome	LAT1 antibody	WP1066	(37)
	liposomes	iNGR	Doxorubicin	(38)
	PEGylated liposomes	Anti-VEGF and anti-VEGFR2 monoclonal antibody	Cisplatin	(39)
	liposomes	Anti-CD133 monoclonal antibody	Gemcitabine and Bevacizumab	(40)
	a cationic liposome	Anti-transferrin receptor single-chain antibody fragments	Temozolomide	(41)
	PEGylated liposomes	Anti-EGFR		(42)
ADC	Monoclonal antibody	The single chain variable fragment (scFv) from the D2C7 monoclonal Antibody (mAb) of EGFR	Pseudomonas Exotoxin PE38KDEL	(43)
	Monoclonal antibody	Monoclonal Antibody against uPARAP/Endo180,	Dolastatin derivative, monomethyl auristatin E	(44)
	Monoclonal antibody	Anti-CD40 agonistic monoclonal antibody (FGK45)		(45)
	Monoclonal antibody	Glioblastoma-specific CD68 antibody	Curcumin	(46)

#### Transferrin Receptor (TfR)

TfR plays a key role in the control of the rate of cellular iron uptake, tuning the amount of iron delivered to the metabolic needs of the cells (47).

The presence of BBB and hard parenchyma of the GBM has been a predominant challenge in chemotherapy in the treatment of GBM. Ever since the finding that iron-loaded transferrin is taken up via receptor-mediated endocytosis at the brain capillaries and transcytosed, many researchers have utilized transferring-transferrin receptor to transfer drugs across the BBB (48).

**Figure 2:**
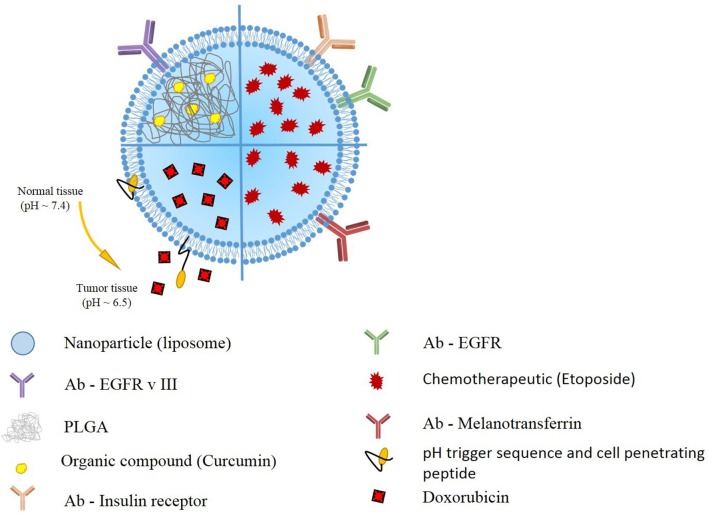
Schematic presentation of selected liposomal nanoparticles. Liposomal nanoparticles are versatile, and can be loaded with wide variety of anti-oncogenic compounds, such as curcumin, etoposide, and doxorubicin). To further enhance the targeting, the outer layer includes antibodies targeting GBM cells, or pH responsive and cell penetrating peptides.

Kuo et al. also utilized this idea to deliver etoposide for the GBM treatment. They generated solid lipid nanoparticles (SLNs) conjugated with melanotransferrin antibody (MA) and examined its transcytosis efficiency across human brain-microvascular endothelial cells (HBMECs) and the resulting growth inhibition of U87MG cells. The *in vitro* transwell assay strategy triggered melanotransferrin-mediated transcytosis and promoted the growth-inhibitory efficacy in U87MG cells suggesting the MA-ETP-SLNs as a promising delivery system for malignant GBM (24) (Figure 2).

The findings that there is a higher reactivity in GBM for anti-TfR and that GBM cells are very sensitive to the effects of anti-TfR mAbs instigated research targeting TfR as a direct way to kill GBM cells rather than a way to bypass BBB (49).

Ramalho et al. developed poly(lactic-co-glycolic acid) nanoparticles functionalized with OX26 type transferrin monoclonal antibody with a purpose to target transferrin receptors on GBM cells (U251 and U87). In this study, the approach facilitated uptake of the nanoparticles by the GBM cells while normal human astrocytes did not internalize the nanoparticles efficiently. However, this encouraging data was not reproduced in comparative cytotoxicity tests with native nanoparticle and TfR-targeting nanoparticle (12).

#### Antibodies for Cancer Stem Cell

Cancer stem cells (CSCs), a small population of quiescent or slowly dividing cells, significantly contributes to the resistance to therapy, and recurrence of cancer. Targeting CSCs could be a good strategy to improve the outcome of cancer therapy. There have also been extensive research to cure GBM through targeting specific markers of CSCs such as CD44, aldehyde dehydrogenase (ALDH) and CD133 as follows.

Mahmud et al. fused human IgG Fc of CD44 with a chlorotoxin peptide (M-CTX-Fc). The authors verified the superiority of M-CTX-Fc by comparing U251MG-P1 cells (CD44+) with CD44-negative cells (SKBR3) in cellular uptake, *in vitro* cytotoxicities and *in vivo* tumor growth inhibition. Since CD44 positivity represent stemness of a cancer cell line along with other markers such as OCT3/4, SOX2, KLF4, and Nanog, this approach may contribute to the retardation of tumor growth by restricting cancer stem cell population (34).

CD133+/ALDH1+ in glioblastoma stem cells (GSCs) were targeted by Kim et al. to deliver Temozolomide with liposome (35). With additional BBB targeting molecule, angiopep-2 (An2), this dual-targeting immunoliposome encapsulating TMZ (Dual-LP-TM) increased *in vitro* cytotoxicity and apoptosis in U87MG GSCs. This approach suggests a potential use of Dual-LP-TMZ as a therapeutic modality for GBM demonstrating significant *in vivo* tumor reduction in intracranial U87MG-TL GSC xenografts (Table 1).

### pH-Responsive Drug Carriers

One of the most widely used intrinsic stimulus for controlled drug release is pH difference between normal tissues and tumor tissue, as well as between cellular compartments. Since tumor metabolism is very active and requires considerable energy for tumor growth, there is increased production of hydrogen ions (H^+^) and lactate resulting in an acidic tumor environment (pH 6.5)(50, 51). Since normal tissue has a pH 7.4, this difference can then be exploited for triggering drug release in the more acidic tumor tissue. Furthermore, the difference in pH between cellular compartments at the cellular level, between endosomes (pH 5.5) or lysosomes (pH 5.0) can be also used to trigger drug release in the cytoplasm. Drug release is usually accomplished by incorporation of an acid sensitive spacer between carrier and drug, which enables drug release at slightly acidic tumor environment or endosomes, and lysosomes of cancer cells.

While these reports use only a pH-triggered drug release mechanism to locally release drug at the tumor site, to further increase specificity Miller at al. (52) constructed a pH-responsive micelle conjugated with a novel moiety against overexpressed cell surface platelet derived growth factor receptor (PDGFR). These micelles are loaded with Temozolomide (TMZ), targeted to PDGFR on glioblastoma cells, resulting in pH-dependent release of TMZ preferably in tumor tissue, thereby reducing systemic toxicity. *In vitro* studies have shown that these micelles exhibit specific uptake and increased cell killing in glioblastoma cells, and *in vivo* studies demonstrated increased accumulation of micelles in brain tumor tissues. Although these results are promising, addition of *in vivo* tumor reduction efficacy and survival experiments would greatly improve the potential of this approach in clinics (Table 2).

**Table 2:** Stimuli responsive targeting macromolecules for GBM treatment.

**Carrier**	**Composition**	**Targeting mechanism(s)**	**Drug delivered**	**Animal model**	**Outcome**	**References**
Polymer based carriers	Albumin	(6-maleimidocaproyl) hydrazone conjugate of doxorubicin	Doxorubicin	U87-luciferase expressing orthotopic xenografts	Aldoxorubicin, U87-luc tumors were 10-fold smaller when compared to control animals, and median survival of Aldoxorubicin treated mice was 62 days, compared to 26 days median survival of control or doxorubicin treated animals	(53)
	Elastin-like polypeptide	Thermo-responsive	c-Myc inhibitory peptide	Rat C6 Glioma orthotopic model	Thermal targeting of the Bac-ELP1-H1 polypeptide to the tumors resulted in significant delayed onset of neurological deficits, 80% tumor volume reduction, and doubled survival.	(54)
	1,2-Distearoyl-sn-glycero-3-phosphoethanolamine (methoxy(polyethylene glycol)-2000) (DSPE-PEG)	pH-sensitive liposomes modified with tumor-specific pH-responsive peptide H_7_K(R_2_)_2_	Doxorubicin	Rat C6 glioma, U87MG human glioblastoma	*In vitro* experiments show pH-triggered DOX release from the pH-sensitive liposomes under acidic conditions The anti-tumor activity has been confirmed in C6 tumor-bearing mice and U87-MG orthotopic tumor-bearing nude mice	(55)
Liposomes	Superparamagnetic iron oxide 1,2-distearoyl-sn-glycero-3-phosphocholine; 1,2-Distearoyl-snglycero-3-phospho-rac-glycerol sodium salt	Magnetic responsive	Doxorubicin	Rat C6 glioma orthotopic model		(56)
	Chitosan-PEG copolymer coated iron oxide nanoparticles, cross-linked and functionalized with BG	Tumor targeting peptide chlorotoxin, redox responsive	MGMT inhibitor *O*^6^-benzylguanine (BG)	Primary GBM6 xenograft tumor model which expresses high levels of MGMT	Treatment with nanoparticles and TMZ showed a 3-fold increase in median overall survival in comparison to TMZ treated and untreated animals.	(57)
Nanoparticles	Poly(ethylene Glycol)-b-poly(trimethylene carbonate-co-dithiolane trimeth-ylene carbonate)-b-polyethylenimine (PEG-P(TMC-DTC)-PEI,	Tumor targeting peptide angiopep-2 (ANG), redox responsive	Protein toxin saporin	U-87 MG-Luc cells orthotopic xenografts	Treatment with polymersomes resulted in 2-fold increase in median overall survival in comparison untreated animals.	(58)
	Human serum albumin (HSA) NPs stabilized with intramolecular disulfide bonds	Tumor targeting peptide substance P (SP) redox responsive	Paclitaxel (PTX)	U-87 MG-Luc cells orthotopic xenografts	The *in vitro* PTX release from NPs occurred in a redox-responsive manner. Treatment *in vivo* showed pro-apoptotic effect and resulted prolonged survival period of treated animals	(59)
	distearoyl phosphoethanolamine-PEG-2000-amine and N-palmitoyl homocysteine	Peptide targeting PDGF receptor, pH-responsive	Temozolomide (TMZ)	U-87 MG-Luc cells orthotopic xenografts	*In vitro* studies have shown that micelles have specific uptake and increased cell killing in glioblastoma cells, and *in vivo* studies reported selective accumulation of micelles in orthotopic glioblastoma model.	(52)
	iron oxide nanoparticles ferumoxytol	MMP-14 activatable peptide, enzyme-responsive	Azademethylcolchicine	pcGBM39 or pcGBM2- orthotopic xenografts	*In vivo* studies demonstrated significant apoptosis of cancer cells and prolonged survival of pcGBM39-bearing mice and complete tumor remission of pcGBM2-bearing mice.	(60)
	poly(ethylene glycol)-poly(ϵ-caprolactone) block copolymer (PEG-PCL)	protamine (LMWP) MMP2/MMP9 activatable peptide, enzyme-responsive	Paclitaxel (PTX)	C6 glioma cells in Orthotopic xenografts in mice	Specific accumulation of PEG-PCL, increased median survival of 48 days when compared to control group (21 days)	(61)

An interesting approach to target glioblastoma, reported by Zhao et al. (55) used tumor-specific pH-responsive peptide H_7_K(R_2_)_2_ as a targeting ligand. This peptide contained the pH trigger sequence polyhistidine H_7_ and cell penetrating peptide arginine rich sequence (R_2_)_2_ and exhibited activity at an acidic pH environment due to the ionization of the histidine thus switching from hydrophobic to hydrophilic conditions. This peptide was used to modify pH-sensitive liposomes loaded with doxorubicin (DOX-PSL-H_7_K(R_2_)_2_). The pH-triggered doxorubicin release from the pH-sensitive liposomes and targeting effect under acidic conditions was demonstrated in *in vitro* experiments. Furthermore, *in vivo* experiments in C6 tumor-bearing mice and U87-MG orthotopic tumor-bearing nude mice confirmed the anti-tumor activity of pH-responsive peptide modified liposomes loaded with doxorubicin. Results showed that the DOX-PSL-H_7_K(R_2_)_2_ (37 days) significantly improved the survival rate of mice compared with control animals (23 days) or doxorubicin treated animals (24 days).

Since doxorubicin is a highly effective anticancer therapeutic for the treatment of many malignancies, there is a great interest in using it in the treatment of glioblastoma. Marrero et al. (53) examined the hydrazine-conjugated doxorubicin derivative, Aldoxorubicin, which binds selectively to Cysteine34 of blood circulating serum albumin, and releases doxorubicin selectively at the tumor site in response to low pH tumor environment. Aldoxorubicin-treated mice exhibited high levels of doxorubicin within the tumor tissue, accompanied by apoptosis of glioblastoma cells and a 3-fold decrease in tumor cell proliferation. Effectiveness of Aldoxorubicin treatment was confirmed in *in vivo* experiments, which demonstrated that when mice were treated with Aldoxorubicin, U87-luc tumors were 10-fold smaller when compared to control animals, or 8-fold smaller when compared with tumors in animals treated with doxorubicin. Importantly, median survival of Aldoxorubicin treated mice was 62 days, compared to 26 days median survival of control and doxorubicin-treated mice. These encouraging results provide a strong rationale to further investigate this approach for the treatment of glioblastoma.

### Redox-Responsive Drug Carriers

Redox-responsive drug delivery carriers exploit the difference in redox potential between the tumor and intracellular environment and normal tissue and blood plasma. Tumor tissues have four times more glutathione (GSH) than normal tissue (62). Furthermore, intracellular concentration of GSH is 3–4 magnitudes higher as compared to the extracellular environment (63). Redox-responsive drug carriers are based on macromolecules containing disulfide bonds which encapsulate drugs. After these redox-responsive carriers are exposed to GSH, disulfide bonds are reduced to sulfhydryl groups resulting in release of encapsulated drugs.

Macromolecular carriers based on different materials, such as proteins, lipids, and polysaccharides have been used as redox-responsive drug delivery systems to target glioblastoma. For example, Stephen et al. (57) developed superparamagnetic iron oxide nanoparticles coated with cross-linked, redox-responsive chitosan PEG copolymers loaded with *O*^6^-benzylguanine (BG). The aim of the study was to selectively deliver BG to the glioblastoma in mice, inhibit the DNA repair protein *O*^6^-methylguanine-DNA methyltransferase (MGMT), and overcome Temozolomide resistance. To further improve tumor targeting, particles were also modified with the tumor-targeting peptide chlorotoxin (CTX). *In vitro* studies confirmed that BG was released from the particles in the reducing environment, and glioblastoma cells were more responsive to TMZ. *In vivo* studies, have shown that treatment with such constructed nanoparticles and TMZ showed a 3-fold increase in median overall survival in comparison to TMZ treated and untreated animals.

In another report, Jiang et al. (58) synthesized redox-responsive virus-mimicking polymersomes (PS) which can efficiently deliver saporin (SAP), a highly potent natural protein toxin, to orthotopic human glioblastoma engrafted in nude mice. To enhance delivery of the drug, polymeromes were modified with angiopep-2 (ANG), a peptide that binds with high affinity to low-density lipoprotein receptor-related protein-1 (LRP-1) which is often overexpressed in glioblastoma cells and brain capillary endothelial cells (64, 65). *In vivo* anti-glioblastoma efficacy experiments have shown that ANG-PS-SAP-treated mice had approximately 7-fold lower tumor bioluminescence intensity than control mice, indicating efficient tumor reduction by ANG-PS-SAP. This was confirmed with 2-fold improvement in median survival time from 22 days in control group compared to 43 days in animal treated with ANG-PS- SAP.

A different approach using human serum albumin (HSA) nanoparticles stabilized with intramolecular disulfide bonds and modified by substance P (SP) tumor-targeting peptide to deliver paclitaxel (PTX) to U87 orthotopic xenografts (59). Animals treated with SP-HAS-PTX nanoparticles exhibited antitumoral effect and prolonged survival time of treated mice when compared to control group.

### Enzyme-Responsive Drug Carriers

Enzymes play important roles in all metabolic and biological processes and dysregulation of enzyme activity and expression is exhibited in many diseases including glioblastoma. Therefore, exploiting overexpression of enzymes and their selective catalytic activity as a trigger to release the drug at the tumor site is a very promising approach.

Mohanty et al. (60) applied this concept to deliver the azademethylcolchicine potent active vascular-disrupting agent. They designed an enzyme-responsive carrier consisting of three main elements: (1) theranostic cross-linked iron oxide nanoparticle backbone, (2) matrix metalloproteinase 14 MMP-14 cleavable linker, and (3) drug azademethylcolchicine. The iron core of nanoparticles enabled *in vivo* tracking of the carrier with MRI imaging, which demonstrated significant accumulation of drugs in the glioblastoma tumors in mice.

Treatment with nanoparticles in combination with Temozolomide achieved tumor remission and increased survival pcGBM2-bearing mice by more than 2-fold compared with treatment with temozolomide alone. Thus, this synergistic combination therapeutic strategy may have significant potential for clinical translation to improve long-term outcomes of glioblastoma patients.

Besides MMP-14, some glioblastomas have upregulated MMP-9 and MMP-2. To exploit increased expression of these proteases, Gu et al. (61) constructed nanoparticles composed of poly(ethylene glycol)-poly(ϵ-caprolactone) block copolymer (PEG-PCL) as the matrix conjugated with activatable cell penetrating peptide protamine (ALWMP, E _10_ -PLGLAG-VSRRRRRRGGRRRR). Positive charges on the LWMP necessary for transduction were at first masked by a polyanionic peptide (E10) via a MMP-2/9-cleavable peptide linker sequence PLGLAG. Once the nanoparticles were exposed to proteolytic activity of the MMPs, transduction activity of cell penetrating peptides was restored. As a result, these particles loaded with paclitaxel (PTX) exhibited elevated MMP-dependent intracellular accumulation in C6 cells, and improved cytotoxicity. *In vivo* imaging demonstrated specific accumulation of the particles in intracranial C6 glioma model in nude mice. Specific accumulation of PEG-PCL nanoparticles in glioblastoma was reflected in increased median survival of 48 days when compared to control group (21 days) or taxol (24) alone. These results are promising, and encourage further *in vivo* experiments in different animal models which would open new modalities for the treatment of glioblastoma based on enzyme-responsive targeted drug release.

### Magnetic and Ultrasound

The integrity of the brain is compromised not only by the highly invasive nature of glioblastoma multiforme tumors, but also further exacerbation occurs with standard surgical resection of the tumor. Surgical resection is followed by radiotherapy and chemotherapy, and efficacy of the therapy is monitored by imaging techniques. There are ongoing efforts in clinics to develop approaches to monitor specificity of the therapy and image the tumor at the same time. These tools are called “theranostics,” and they integrate imaging and therapeutic modality in the singe macromolecule. Wide application and versatility of theranostic complexes have led to design and production of various different theranostic compounds.

To use the maximum potential of such theranostic compounds, several groups have included ultrasound in their methodology. Effective drug delivery to the brain tumor is primarily hampered by blood-brain barrier and to overcome BBB, it has been reported that focused ultrasound (FUS) can be used for temporarily opening of the BBB (66, 67). One such approach was used by Fan et al. (56), 2016 for local drug delivery in a rat glioma model. The group has fabricated Superparamagnetic iron oxide (SPIO) conjugated with doxorubicin and embedded in lipid microbubbles (MBs), namely SD-MBs. SD-MBs compounds were used for augmented drug delivery to the brain tumor. The animals underwent FUS sonication after bolus injection of SD-MBs, with the purpose of opening BBB and easier tumor perfusion. The FUS sonication was followed by magnetic resonance imaging (MRI) for SD-MBs visualization, simultaneously with magnetic targeting (MT) for increased drug delivery to the tumor site.

Although theranostic tools are very promising and versatile, future studies should be further focused on efficiency of tumor reduction and survival in glioblastoma animal models as well as treatment safety. These more extensive preclinical studies would justify applying this approach in future clinical treatments.

Similar strategy has been used in another study with thermo-responsive liposomes (68). Liposomes were modified with gadolinium and rhodamine and could therefore be used for both ultrasound-mediated drug delivery as well as MRI and optical imaging. The group synthesized t liposomes with different transition temperature (Tt), the temperature at which liposomes undergoes gel-to-liquid crystalline phase transition. One thermoresponsive liposome, The New Thermosensitive liposome (NLP), was designed with a Gadolinium–DOTA lipid bilayer and a Tt of 42°C. The second thermosensitive liposome The Conventional liposome (CLP), was designed with Gd- DTPA-BSA lipid and a Tt of 60°C (68, 69). At determined Tt the transmembrane permeability of liposomal complex was increased.

Using light microscopy to show that the designed liposomes accumulated in the flank of a murine glioma model, they further modified the liposome surface with biotin and rhodamine, which tightly binds to Gli36 glioma cells expressing biotin acceptor peptide (BAP). Significantly higher accumulation of liposomes was observed in BAP-expressing tumors, indicating efficient tumor targeting and imaging capabilities using MRI.

Since the designed liposome are thermo-responsive they have a potential to be targeted to the tumor tissue and release the drug when external mild heat is applied. To further demonstrate drug delivery potential, additional experiments including drug encapsulation and determination of stability of liposomes in plasma and efficacy in orthotopic glioma model are necessary to advance this technology to its full potential.

### Temperature-Responsive Drug Carriers

Thermo-responsive drug delivery carriers are one of the most investigated stimuli-responsive strategies for targeted, stimuli-responsive drug delivery. Temperature-responsive drug carriers undergo phase transition and rapid change in their physical property at certain temperatures; lower critical solution temperature (LCST). Below LCST, drug carriers are soluble but upon heating they become insoluble, which may increase drug carrier accumulation or trigger drug release in the heated tumor area. Moreover, LCST may be modulated by incorporation of hydrophilic or hydrophobic monomers to achieve LCST temperature corresponding to mild hyperthermia (37–42° C). This temperature range is desirable, since it is higher than normal temperature, but lower than temperatures which may damage healthy tissue. Furthermore, mild hyperthermia can be effectively localized and contained within the tumor site without spilling into adjacent normal tissue. As tumors have a defective vascular architecture and impaired lymphatic drainage, the application of mild heat results in the preferential retention and increased concentration of drugs. Additionally, hyperthermia is a mature clinical modality currently used in clinics, rendering the methods, and techniques necessary to employ targeting of thermally sensitive polypeptides available in the clinical setting. Further hyperthermia increases blood flow, resulting in an increased permeability of the tumor, as compared to normal vasculature and hyperthermia increases tumor vasculature pore size, enhancing extravasation of macromolecules (70, 71) and cellular uptake (72, 73).

#### Elastin-Like Polypeptides

**Figure 3:**
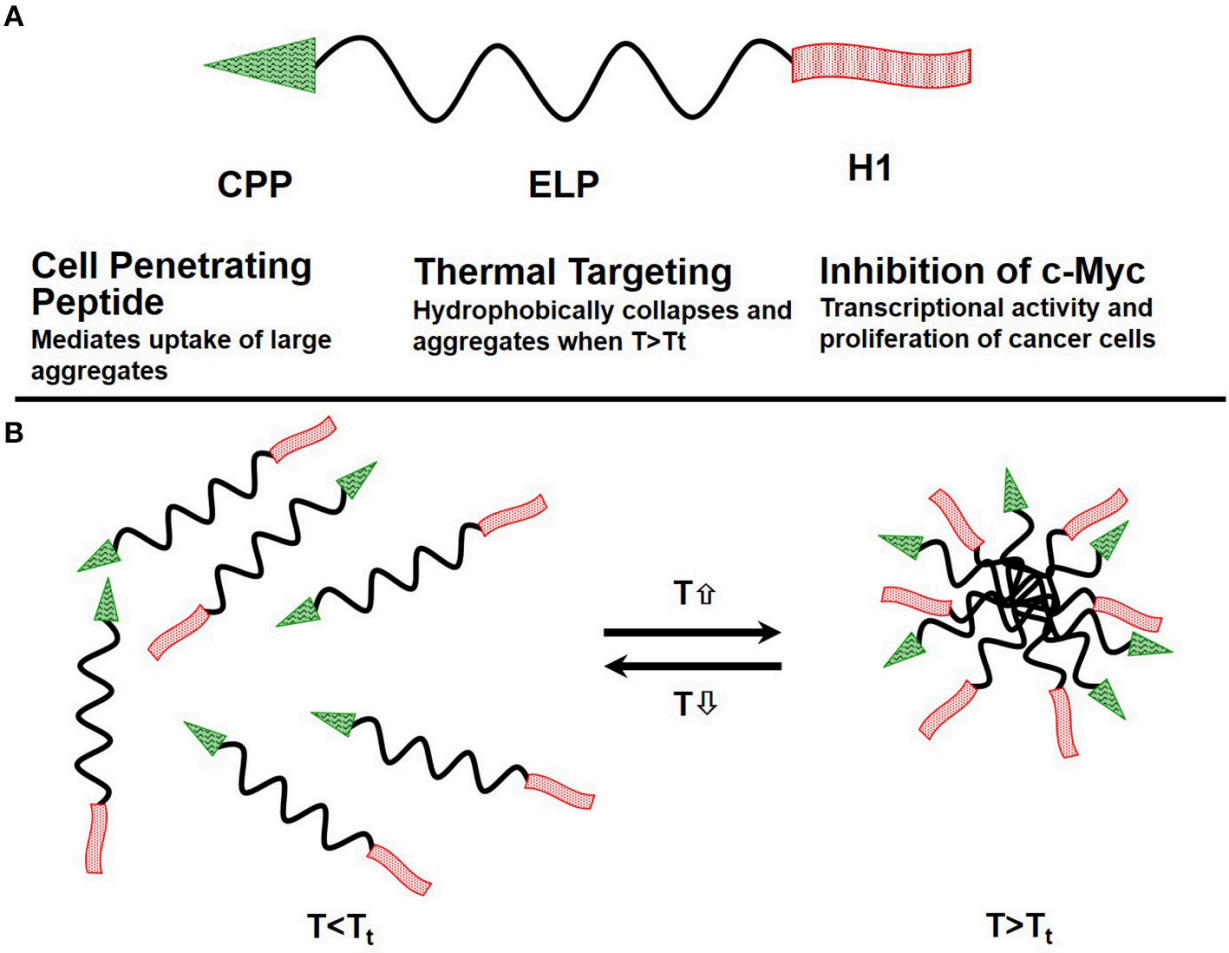
Schematics of the ELP-based drug delivery vector**. (A)** The delivery system consists of the cell penetrating peptide (CPP) Bac, which promotes cellular uptake of the polypeptide, the thermally responsive elastin-like polypeptide, and a c-Myc transcriptional activity inhibitory peptide (H1), which inhibits cancer cell proliferation. **(B)** ELP remains a soluble monomer when the solution temperature is at or below body temperature. When solution temperature is raised above body temperature T > T_t_, it hydrophobically collapses and forms aggregates.

One class of thermo-responsive drug carriers, which was developed in our lab, is based on the thermally responsive biopolymer elastin-like polypeptide (ELP). An ELP, soluble at physiological temperatures, undergoes a phase transition and aggregates in response to an externally applied mild hyperthermia (40–41°C). Our ELP's coding sequence was modified by adding a cell penetrating peptide (CPP) Bac, to enhance polypeptide delivery across the blood brain barrier (BBB) and to facilitate cell entry. Also added was a peptide, derived from helix 1 (H1) of the helix-loop-helix region of c-Myc (H1-S6A, F8A), to inhibit c-Myc transcriptional activity and cell proliferation. Schematic of the ELP based drug delivery vector was presented in Figure 3.

ELPs are genetically engineered biopolymers that, in addition to all the benefits of macromolecular drug delivery systems, provide a number of additional advantages: (1) ELPs are thermally responsive biopolymers which undergo a sharp (2–3°C range) phase transition, leading to desolvation and aggregation of the biopolymer when the temperature is raised above their Tt (74, 75), rendering them suitable for thermal targeting; (2) ELPs are genetically encoded, providing control over the ELP sequence and molecular weight (MW) to an extent impossible with synthetic polymer analogs which allows ELP molecular weights to be precisely specified, resulting in monodisperse polymers, a feat difficult to achieve with synthetic polymers; (3) ELP composition can be encoded at the gene level, allowing an ELP sequence to be modified by adding cell penetrating peptides, and therapeutic peptides. These targeting peptides can then be used to define tissue distribution, tumor penetration, and sub-cellular uptake/localization. Together, these properties make ELPs a promising class of biopolymers for targeted drug delivery.

##### Thermally targeting increases delivery of CPP-ELP1-H1 to intracerebral gliomas

**Figure 4:**
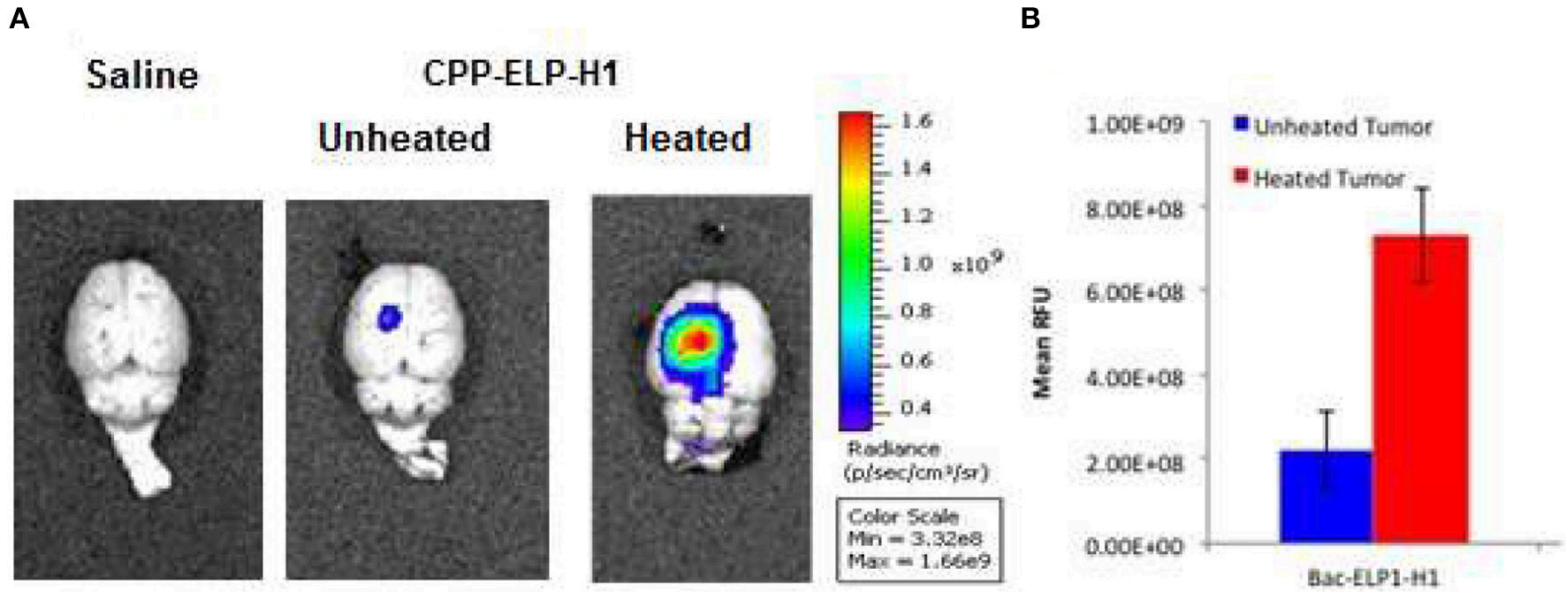
Enhancement of CPP-ELP1-H1 Tumor Uptake by Thermal Targeting. Following IV administration of Alexa750-labeled CPP-ELP1-H1 with or without hyperthermia, construct levels in tumors and organs were determined by *ex vivo* whole organ fluorescence imaging. **(A)** Representative brain images from each treatment group. **(B)** Quantitation of tumor fluorescence from each group. Bars, s.e.m. *, Fluorescence levels differ statistically (*p*, 0.01, one way ANOVA with *post-hoc* Bonferroni; *n* = 4 rats/group).

We tested the ability of the CPP-ELP1-H1 polypeptide to be thermally targeted to tumors. Rats bearing intracerebral tumors were injected IV with Alexa750-labeled BacELP1-H1. Tumors were heated using the described thermal cycling protocol (54), and tumor deposition was determined by *ex vivo* imaging of rat brains 4 h after injection using an IVIS Spectrum animal imager. Polypeptide accumulation in tumors occurred at a high level relative to adjacent normal brain (Figure 4A). Moreover, tumor polypeptide levels noticeably increased when Bac-ELP1-H1 treatment was combined with tumor hyperthermia. Quantitation of tumor fluorescence intensity revealed that thermal targeting increased Bac-ELP1-H1-Alexa750 tumor accumulation by 3.3-fold (Figure 4B, *p* = 0.0004, Student's *t*-test).

##### CPP-ELP1-H1 can penetrate the BBB and enter GBM cells

**Figure 5:**
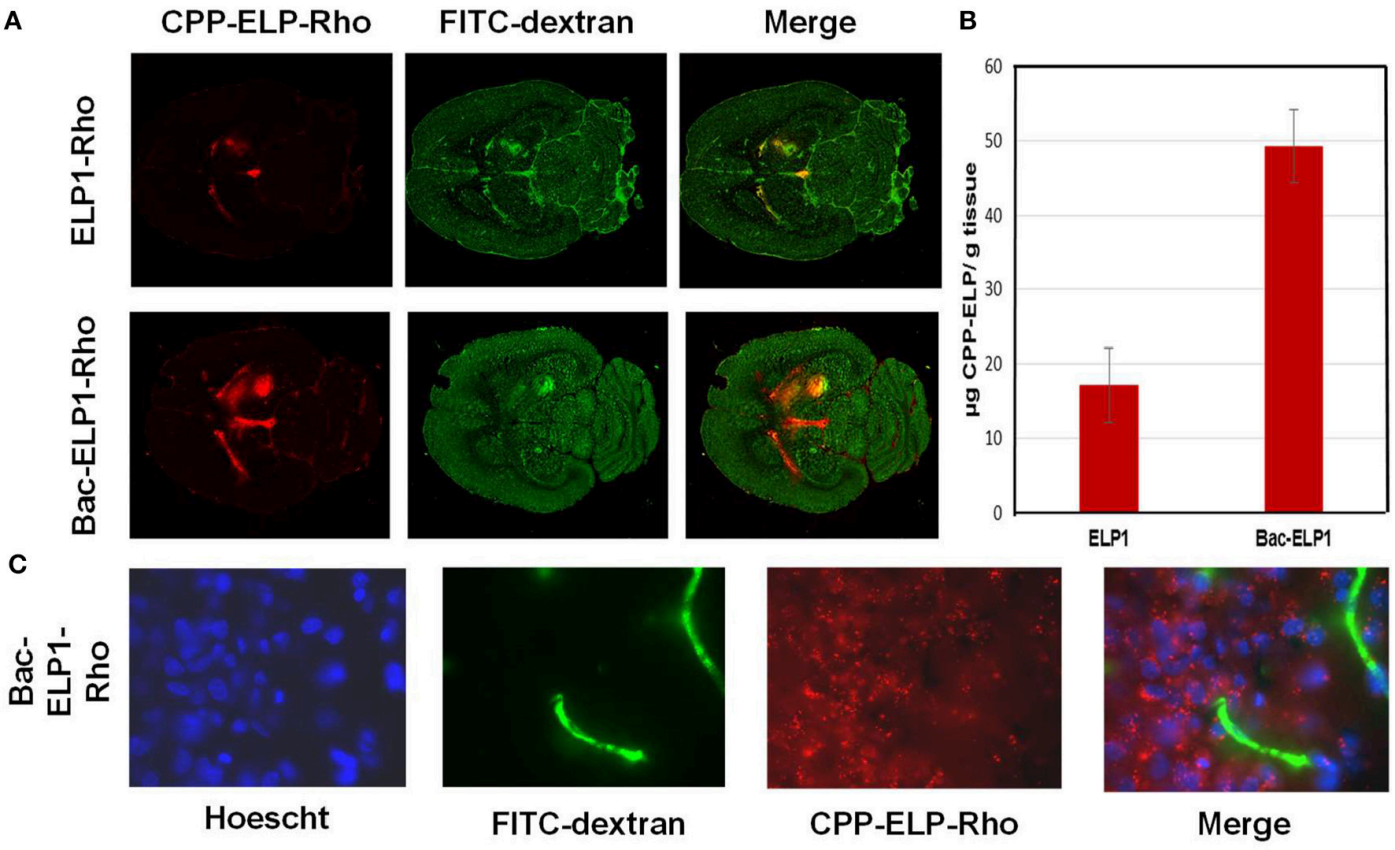
Tumor and Cellular Uptake of CPP-ELP. **(A)** Distribution of rhodamine-labeled polypeptides in tumor and normal brain relative to perfused vasculature. Rhodamine fluorescence was used to follow the localization of the polypeptide within the tumor (left panel); the perfused vasculature was marked by infusion of high molecular weight dextran 1 min prior to euthanasia (middle panel). **(B)** Tumor levels 4 h after IV administration of rhodamine-labeled ELP or CPP-ELPs. Bars, s.e.*, Tumor levels are significantly enhanced (*p*, 0.01, one way ANOVA with *post-hoc* Bonferroni, *n* = 6 rats/group). **(C)** Microscopic images of tumor sections were collected after staining cell nuclei with Hoechst 33342 using a 60 X magnification objective.

A major barrier to GBM treatment is posed by the BBB, which any proposed therapeutic must penetrate. To assess the ability of CPP peptides to do so, and to determine their capacity to mediate ELP drug carrier delivery into C6 brain tumors, rats bearing intracranial C6 tumors were IV injected with Rhodamine-labeled CPP-ELP1 or an ELP1 control. At 4 h after injection, a 500 kDa FITC-dextran was injected to mark perfused vasculature, the animal sacrificed, and the brain removed, frozen, and sectioned (Figure 5A). Slides were scanned with a ScanArray Express slide scanner (Perkin Elmer), with fluorescence intensity determined using Image J software. Tumor intensity, expressed relative to plasma concentration at time zero (RFU/C_0_), was averaged for all animals. ELP passive accumulation in C6 tumors was higher than in normal brain tissue; however, adding a CPP greatly enhanced tumor fluorescence relative to unmodified ELP. Comparing CPP-ELP-Rhodamine fluorescence with FITC-dextran fluorescence (Figure 5B) showed: (1) slightly greater enrichment of perfused vessels in tumors than in normal brain, and (2) significantly greater polypeptide accumulation in the tumor than in normal neural tissue. Microscopic examination of tumor sections showed the presence of CPP-delivered ELP in the blood vessels, extravascular space, and within tumor cells (Figure 5C). These data indicate the ELP polypeptide's passive accumulation in brain tumors in this rat model, as well as the enhancement conferred for total tumor levels and deposition throughout the tumor, relative to a non-CPP containing control, by using the CPP.

##### Reduction of intracranial C6 tumor proliferation by Bac-ELP-H1

After demonstrating that CPP-ELP-H1 can enter C6 tumors in brain, the construct's effects on tumor progression and animal survival were evaluated.

Rats bearing intracerebral C6 tumors were treated daily for 4 days beginning on day 9 after implantation. The CPP-ELP1-H1 polypeptide, or control polypeptides lacking the H1 peptide (Bac-ELP1) or utilizing the non-thermally responsive version of ELP (Bac-ELP2-H1), was injected IV. In the hyperthermia groups, hyperthermia was applied to the tumor site using a thermal cycling protocol immediately after each injection. Tumor progression was monitored using multi-slice 3D T1 trans-axial

**Figure 6:**
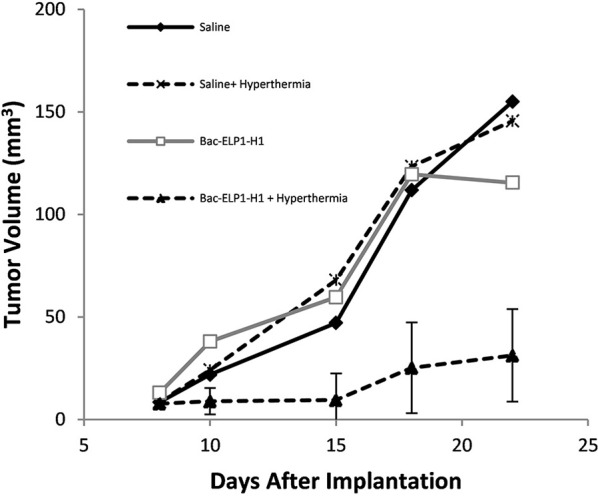
Inhibition of Glioma Progression by the Thermally Targeted c-Myc Inhibitory Polypeptide. Sprague Dawley rats bearing intracerebral C6 tumors were treated with 4 daily IV injections of the Thermally Targeted c-Myc Inhibitory Polypeptide, with MRI monitoring of tumor volume. Average tumor volume for each treatment group. *n* = 6-8 animals per group; *, Tumor volume was significantly reduced compared to control tumors (one way ANOVA, *post hoc* Bonferroni).

Imaging with a gadolinium-based contrast on days 10, 15, 18, and 22. As shown in Figure 5, the C6 tumors progressed rapidly in all treatment groups except those in the Bac-ELP1-H1+ hyperthermia group; in this group, tumor volumes were 80% smaller, with a mean volume of 31 mm^3^ (*p* = 0.004, one-way ANOVA, Figure 6).

Control polypeptides without H1 peptide (CPP-ELP1) had no effect on tumor reduction, while the non-thermally responsive ELP (CPP-ELP2-H1) resulted in a 30% tumor reduction (data not shown).

These results are significant, since they demonstrate that it is feasible to increase brain tumor uptake of thermally responsive ELP drug carriers with focused hyperthermia, but also thermal targeting of the Bac-ELP1-H1 polypeptide to the tumors resulted in significant delayed onset of neurological deficits, 80% tumor volume reduction, and at least doubled survival.

While these results demonstrate that use of ELP to thermally target the H1 peptide, similar approach may be used to apply ELP technology for delivery of other therapeutic peptides for glioma. Future studies should expand this testing into other GBM models, including mouse orthotopic xenografts of human glioblastoma cells.

## Conclusion and Future Perspectives

The current treatment of glioblastoma is particularly challenging not only because of the delivery of therapeutics to the brain, but also because of the tumor heterogeneity, aggressiveness, and recurrence. Although prognosis for the glioblastoma patients remains poor, recent developments in drug delivery approaches provide hope for the successful treatment of glioblastoma. This article reviewed recent progress and potential of macromolecular drug carriers. Macromolecular carriers increase efficacy, stability and plasma half-life of anticancer drugs, and reduce toxicity to healthy tissues. Tumor targeting of macromolecular carriers mostly rely on the passive tumor targeting via the enhanced permeability and retention effect. However, in addition to passive targeting, numerous macromolecular carriers have been developed to deliver and/or release drugs in response to internal or external stimuli, including pH, enzymes, redox potential, magnetic field, ultrasound, and temperature. These stimuli-responsive macromolecules provide active targeting for anticancer drugs and further improve delivery of the drugs specifically to the tumor tissue. However, despite the progress which has been achieved in development of macromolecular carriers, some challenges for their successful clinical application remain.

Beside heterogeneity of tumors across the patients and tumor types, such as difference in pH and expression of specific enzymes, both of which may influence drug delivery in response to internal stimuli, there is also the issue of non-specific biodistribution of macromolecular carriers in other organs, such as liver and kidneys. Furthermore, complex design of some of the carriers and difficulties in scaling up their production may present further limitations in clinical applications. Due to these reasons there are only a limited number of macromolecular carriers presently used in clinics. Substantial progress may be possible if the research efforts are also focused not only on developing efficient macromolecular carriers, but also on development and selection of clinically-relevant animal models and assays which can more precisely predict their potential toxic effects.

## Author Contributions

DR wrote the initial drafts of the manuscript. SD and JR wrote subsequent drafts of the manuscripts, and produced figures. All authors reviewed and approved the final submitted manuscript.

### Conflict of Interest Statement

DR is the president of Thermally Targeted Therapeutics, Inc., Jackson, MS, USA. The remaining authors declare that the research was conducted in the absence of any commercial or financial relationships that could be construed as a potential conflict of interest.
